# Clinical Lectures on Certain Diseases of the Urinary Organs

**Published:** 1857-11

**Authors:** 


ART. III.—*Clinical Lectures on Certain Diseases of the Urinary Organs, and on Dropsies.* By ROBERT BENTLEY TODD, M.D., F.R.S., Physician to King's College Hospital. London: John Churchill. Reprinted by Blanchard & Lea. 8vo. pp. 283. 1857.

To notice the productions of able medical authors is always a pleasant task; the more pleasant, if to the reputation for ability they add that of candid and indefatigable observers. The volume before us emanates from one whose contributions to science for many years back, have attested both ability and conscientious industry of so high an order, as to render the perusal of anything coming from him, on a subject so difficult clearly to expound as clinical medicine, a source of positive enjoyment.

The first collection of these lectures treated of the diseases of the nervous system. This second series is intended to elucidate dropsies and certain diseases of the urinary organs, and is appropriately inscribed to William Bowman, F.R.S., a name closely connected with their anatomy, and more than once before associated, for the good of science, to that of Robert Bentley Todd.

We trust sincerely that these volumes will be continued. There are many, too many works on the practice of medicine, with mere general descriptions, and far too few in which the symptoms and the treatment of individual cases are discussed. We need more detailed clinical reports in our works on practical medicine. A few such volumes as Andral's Clinic, Graves's Clinical Lectures, Watson's Lectures, made up or illustrated, as they are, by cases well observed and as well described, are of more advantage to the profession and tend more to the advance of our knowledge of diseases than hundreds of text-books containing only general statements. The latter are undoubtedly instructive; but the former are instructive too, more interesting, more forcible; and furnish, in addition, valuable matter, which, stored away now, will gradually accumulate, to be used by future investigators, for the better understanding of present, and the elaboration of new principles in our art.

The first lecture of Dr. Todd is devoted to the subject of *hæmaturia.* This, it is well stated, is not a disease in itself, but a mere symptom, a mere occurrence of blood in the urine. Urine containing blood is either of a light red, or, as is more general, of a dark smoky color, and yields a deposit by heat and nitric acid. The microscope may save the necessity of subjecting it to chemical test, for it determines the presence of blood by exhibiting the blood-corpuscles.

The presence of blood being settled, it becomes of importance to ascertain its source. The hemorrhage may come from the kidney, from the bladder, or from parts adjacent to the urinary apparatus. Nothing but a careful attention to all the symptoms will indicate the organ at fault. If from the bladder, local pain on pressure and more or less difficulty in micturition occur; the contents voided are clear at first; the last portion is red and apt to contain clots. If the kidney be the source whence the blood is derived, there will be pain in the lumbar region, and usually an absence of clots from the urine, the blood being uniformly diffused through the secretion. The microscope again may aid us, to some extent, in a differential diagnosis. In hæmaturia of renal origin, globular epithelium and casts of the renal tubes imbedding blood-corpuscles, and epithelial cells are met with; yet these are not always present, as the first case reported proves.

Hæmaturia may be associated with inflammation of the kidney, with scarlatinal dropsy, with rheumatism, with gout, with renal calculi, with impoverished state of the blood, inflammation of the ureter, and with other morbid conditions, which Dr. Todd fully notices in the first two lectures.

The cause of the hemorrhage is in several cases explained by what we shall, for want of a better term, call the "eliminative" theory. To those of our readers who have perused the recently published works on the diseases of the kidney, this view will be familiar; to those who are unacquainted with it, we would say, that it is assumed that in certain diseases, amongst which scarlatina, gout, and rheumatism hold the foremost rank, morbid material has to be ejected from the system. To effect this, a heavy task is imposed upon the glandular structures, and especially upon the kidneys: they become thus irritated, engorged, inflamed, their delicate vessels rupture, and blood is mixed with their proper secretion. It is not for us here to discuss this view of elimination, which is, after all, but the revival, in a more scientific form, of the old doctrine of peccant humors, applied to explain the morbid state of individual organs. Whether we adopt it or not, it is certain that in some diseases in which it may be presumed that a morbid material is retained in the blood, as where the action of the skin is impeded by cold and exposure, or in scarlet fever, an inflamed or irritated state of kidney follows. This again leads to acute dropsy and to bloody urine.

The treatment of hæmaturia thus caused, consists in relieving the active irritation of the kidney by remedies addressed to it, in restoring the full action of the skin, and in ridding the system of the amount of water accumulated in its cavities and areolar tissues. Cupping the kidney, hot baths, and purgatives, would seem to be indicated, and are, for the most part, resorted to. The former remedy does not always meet with hearty approval.

"With the hope of relieving the active congestion of the kidney, our patient was cupped over the loins, and several ounces of blood taken away. I cannot say that he derived any benefit from this; and, I must confess, that in the treatment of similar cases I have been more frequently disappointed than satisfied by topical bloodletting, when the congestion of the kidney was active. I suspect that as long as the morbid matter is undergoing elimination through the kidney, and keeping up irritation of the gland, local bloodletting does little or no good. If a particle of dust gets into the eye, it excites conjunctival inflammation; you may leech the eye, day after day, until your patient is blanched; yet active congestion of the conjunctiva will continue; but remove the particle of dust, and the congestion will quickly subside. So with the kidney;—you will do more to relieve the active congestion of which it is the seat, by opening new channels for the elimination of morbid matter, restoring and promoting the action of the skin, and increasing that of the bowels, than by the withdrawal of blood. But when these evacuations have been some time in action, and the congestion of the kidney has assumed a passive character, then the removal of blood by cupping, or by leeching, will often succeed in relieving the congestion."

After the hyperæmic state of kidney has subsided, and as the kidney is resuming its secreting action, Dr. Todd recommends the bitartrate of potassa in diuretic doses. Its exact action does not seem clear to him, yet although he advances a theory to justify its administration, he adds, in a spirit of true philosophy, that "experience tells so much in its favor," that we should not be justified in abstaining from employing it, merely on account of an hypothesis, which may or may not be well founded.

When the hemorrhage has been of long duration, even if owing to inflammatory action at the onset, the blood becomes poor, and the nutrition of the patient falls off. (Cases III and V.) Under such circumstances, the hemorrhage becomes passive, and is kept up, Dr. Todd thinks, by a rupture of the ill-nourished and weakened vessels. Antiphlogistic and diuretic remedies must then be abandoned, and stimulants, astringents, and nutritive diet take their place. Port wine and gallic acid are especially useful.

We have thus seen that hæmaturia is a frequent symptom of inflammatory engorgement of the kidney. This may itself again be the result of exposure to cold, of scarlet fever, or of other states, in which a poisonous substance is presumed to be retained in the blood. The seat of the disease is in the uriniferous tubes. Dr. Todd describes a second form of inflammation, with which bloody urine may be associated. He calls it phlegmonoid. "It is like the phlegmonoid inflammation elsewhere," passes rapidly into suppuration and sloughing, destroys the tissues it involves, and leaves pus-secreting cavities, which cause pus, mixed with blood, or succeeding this, to be found in the urine. It may be occasioned by a renal calculus, by blows on the kidney, or may occur in the subcutaneous areolar tissue in an inexplicable way, and much as a furuncle or carbuncle is formed. It generally attacks only one kidney. We find tenderness on deep pressure, but the attack is not traceable to any special exposure, nor is it associated with dropsy. The fever accompanying phlegmonoid nephritis is of the intermittent or remittent type. If, as is stated, its main character is, that it passes rapidly into the suppurative process, we confess we do not see in this any very strong analogy to a boil on the skin, nor indeed does it appear why the term phlegmonoid nephritis should be preferable to the ordinary one, of acute suppurative nephritis.

Hæmaturia is very commonly (Dr. Todd says most commonly) the result of the irritation of *renal calculi.* There is pain in such cases referred to the kidney and the course of the ureter. Dr. Todd cites, however, some interesting cases to prove that pain may exist in these regions, and other symptoms of renal calculi be met with, without the presence of these bodies, the real lesion being inflammation of the ureter or malignant disease of the kidney.

Several lectures are devoted to those diseases which are associated with *an albuminous condition of the urine and with dropsy.* Since the great discovery of the celebrated physician, who pointed out the connection between this group of symptoms with morbid changes in the kidney, that organ has been repeatedly examined with care, to ascertain exact lesions and their causes, but alas! although much has been done, more remains undone. Dr. Bright originally admitted three stages of the disease which bears his name; Rayer soon more than doubled the number. Of the more recent observers, Frerichs delineates three stages; on the other hand, some pathologists, at the head of whom we find Johnson and the author of the work before us, multiply the honor due to Richard Bright, by denying the *unity* of the affection he described, and contending for its being a *group* of diseases, including, not several stages of one malady, but affections which it is proper to disconnect and consider apart, as they have; but this feature, invariably, in common,—the existence of more or less albumen in the urine.

It is thus that we have the acute and chronic desquamative nephritis, the fatty kidney, the waxy kidney, the gouty kidney, and so on. These, studied in a clinical point of view, Dr. Todd tells us, present themselves in two classes: one, which comprises the cases in which the dropsy is decided, and occurs as an early symptom; and the second, in which it is variable and usually slight.

The first form is the well-known *acute* dropsy, the result of exposure to wet and to cold, or observed as one of the many ills that render an attack of scarlet fever a source of such uneasiness to the practitioner. When dropsy takes place under such circumstances, the kidney becomes enlarged, sheds its epithelium, which is seen on the fibrinous casts of the tubules, along with blood-corpuscles in the heavy, dingy urine voided by the patient.

This is the disease called by some acute Bright's disease, by others acute desquamative nephritis. The dropsy after scarlet fever may be, perhaps, best taken as its type. The symptoms are a dry, harsh, rough skin, abundant dropsy, and dense, highly albuminous urine. The effusion commences mostly in the eyelids and face, and then spreads over the surface of the body, involving often some of the serous membranes. The internal cavities may, however, be the first to suffer, as proved by the interesting case of Rilliet,* in which a boy of five years of age was seized with a double hydrothorax after scarlet fever. The urine was bloody and highly albuminous; yet it was only after the effusion in the chest was gradually absorbed, that the face and limbs commenced to swell.

The dropsy appears generally in the third week after the eruption of scarlet fever; it may appear earlier, it may not occur until eight weeks after desquamation. Slight cases are more liable to it than those in which the eruption has been very extended and the desquamation complete. In its train it brings impoverished blood, a tendency to serous inflammation, and to uræmic poisoning.

Dr. Todd conveniently accounts for the impoverished state of the blood by the poison of scarlet fever interfering with the proper development of the red particles, and pursuing this idea further, argues that the capillary circulation is disarranged by the presence of the poison, thus still more favoring effusion. The blood is filled with water; the kidneys, irritated, are unable to excrete the fluid; it must find a vent, and permeates the areolar tissues of the body, mainly those of the skin, on account of the determination of blood to it, caused by its irritated condition.

The occurrence of dropsy after scarlet fever is fraught with danger, excepting in those cases, in some epidemics, and in some countries the most usual, in which dropsy exists without albuminous urine. To this class belonged those sixty cases spoken of by Dr. Phillips, of Berlin, in all of which recovery took place. Of the general prognosis in England, and we suppose it is about the same in this country, we may judge from the cases collected by Tripe.† In 11,566 fatal cases of scarlatina, occurring in the five years, 1848 to 1852, were included 1600 from dropsy, being in the ratio of 13·8 deaths from dropsy in each hundred from scarlatina. It is presumable that an albuminous state of the urine existed in all these cases. Yet even under such circumstances recoveries, we are told by Dr. Todd, are, if the patient be treated with care, not unusual; nor does he believe that patients thus affected preserve any special liability to renal disease, in other words, he withholds his sanction from the view, that

* Rilliet & Barthez, p. 175, vol. ii.

† Scarlatinal Dropsy, by John W. Tripe, M. D., Brit. and For. Med. Chirurg. Review, 1854.

the acute desquamative nephritis of scarlet fever predisposes or ends in the chronic forms of Bright's disease. The dropsy, if the case is to terminate favorably, disappears gradually or suddenly; if an unfavorable termination is to occur, it is apt to remain stationary, or to increase. The immediate cause of the fatal result may be the extreme impoverishment of the blood above alluded to, or pulmonary œdema complicated with pericarditis, or extensive inflammation of other serous membranes. Uræmic poisoning, again, may occasion the most frightful convulsions, or a low comatose condition. Several interesting cases of this kind are reported in Lectures VI and VII.

The treatment in cases of acute dropsy is obvious. Spare and soothe the kidney; keep up the action of the skin and bowels. Warm baths, purgatives, and mild diuretics, such as the liquor ammoniæ acetatis, the bitartrate of potassa, diluents, rest, are remedies to fulfil these indications. In cases complicated with bronchitis, antimony is very useful. The diet must be nutritious and easy of digestion. Wine may be administered to support the patient's strength. Mercurials are too lowering; they are, in all cases, to be guarded against; so are irritating diuretics, as squills or cantharides. Bloodletting, especially general, requires great caution.

Dropsy, accompanied by the presence of albumen in the urine, may occur in a gradual way. This *chronic* Bright's disease is spoken of by Dr. Todd under different names in his second group of cases. The dropsy is usually slight. The urine contains albumen in varying quantity; and with the varying quantity of this substance, the specific gravity falls to 1010 or rises to 1035. These different conditions may be found associated with at least two different pathological states. The kidneys are larger, or else they are smaller and harder than the normal organs. Many who perfectly admit these pathological differences, see in them but successive stages of the same morbid state. Not so Dr. Todd. He not only separates them pathologically, but also clinically. Nay, further, going almost to the extent of that champion for subdivision, Dr. Johnson, he makes mention, under chronic enlargement, of two kinds "sufficiently distinct to be diagnosed;" one being that which first attracted the notice of Dr. Bright, the large mottled kidney, the peculiar features of which the discoveries of late years have shown to depend upon the deposition of fatty matter in the epithelium of the uriniferous tubes; the second, when the enlargement is due to the deposit of a waxy-looking fibrinous material, occasioned by what has been termed waxy degeneration.

The *fatty* kidney occasions most of those symptoms which we are in the habit of attributing to chronic Bright's disease. In it we have the pale, puffed face; the obstinate and increasing dropsy; the bronchial irritation and dyspnœa; frequency of micturition, and a large deposit of albumen induced by heat and nitric acid. The sediment in the urine exhibits many fatty casts, fat cells, and free oil. Now, in the other form of the enlarged kidney, or the *waxy* disease, there is no dropsy, or it is trifling in amount; the face is not swollen, but sallow and thin. Emaciation is, indeed, for the most part, a marked symptom. The urine contains much albumen, but only a few casts, which are pale and transparent. These cases usually occur with similar waxy enlargement of the liver and spleen. They may be of long duration, and are to be regarded, according to the author we are noticing, as the result of a general cachexia, allied, perhaps, to scrofula.

Exactly the opposite, in an anatomical point of view, and presenting, it is here stated, marked clinical differences, is the small, dense, contracted kidney. From its very frequent occurrence in persons of a gouty diathesis, Dr. Todd proposes to call it the *gouty* kidney. The symptoms by which this contracted or gouty kidney can be recognized, are, in the main, those of the other form of chronic renal disease. But as the disease advances, a greater tendency to coma, and to convulsions, exists, on account of the kidneys losing their power to eliminate urea and other obnoxious matter. The blood becomes thin and impoverished, and charged with urinary constituents. Epistaxis is frequent. The urine is pale, and of a low specific gravity; its quantity is not diminished. It contains but little albumen, many large granular casts of the uriniferous tubules, altered epithelium, and a few waxy casts and fat-cells. Dropsy is present, although it is not as abundant as in the fatty kidney; but more so than in the waxy disease. The face is sallow. The patient is, according to Dr. Todd, usually of a gouty diathesis; in his joints there may be proofs left of the ravages of the disease. The liver is apt to suffer and become contracted; the heart diseased. In cases presenting these complications, more dropsy, especially in the peritoneum and lower extremities, may exist. Such is the outline of symptoms we have been enabled to group together from the cases of "gouty" kidney mentioned in these clinical lectures. It will be observed that they vary somewhat from those of the other form of chronic renal disease, which give rise to the enlarged kidney. The manner in which the contracted kidney is produced, is stated to be by the continued irritation of the poison of gout, or one akin to it, which, by creating a highly disturbed state of nutrition, leads to atrophy; that it is the result of inflammation is denied.

Now, the relation to the gouty diathesis seems to us to have been overrated. In many countries, rheumatism is not very frequent, and gout extremely rare; yet the writers of these countries do not mention cases of contracted kidney as anything so much rarer than the other pathological states of renal disease. Seeing so many people, as does Dr. Todd in the city he lives in, afflicted with this strange and singularly painful malady, it is, perhaps, not surprising that his mind should have dwelt much on its varying phenomena, and have sought to bring organic lesions and pathological changes in connection with it. That such is the case may be judged from a sentence in the twelfth of these collected lectures: "A knowledge of the real nature of gout, and of its kindred malady, rheumatism, is, in my opinion, at the very foundation of all sound pathology." Is the pathology of inflammation, of tubercle, of cancer, not as important? Are gout and rheumatism at their foundation?

With regard further to the production of these contracted kidneys, mainly by a process of atrophy, and not as the result of inflammation, it strikes us as strange that the elimination of one poison, as of scarlet fever, should occasion congestion and enlargement, and that of gout or a diathesis akin to it invariably a dwindling without inflammation. But from Dr. Todd's own statement in the very first case described in the book, it follows that an actively engorged state of the kidney may occur in the elimination of the rheumatic poison. If in rheumatism, why not in gout? stated all through the book as the same diathesis. If a state of active irritation with hæmaturia from rheumatism, why not a state of inflammation, with gradual change in the effused lymph from gout? Or are the boundaries between active hyperæmia and inflammation so distinct, that in an organ where one occurs, the other may not be presumed to follow?

The gouty or contracted kidney is the last form of renal disease, connected with dropsies, mentioned. We subjoin a tabular form for their diagnosis, enlarged from that printed at the end of the third lecture. We have added the differences of the state of kidney, and the main symptoms and diagnostic facts, as we have been following them out.

A.

*Cases in which dropsy is urgent and acute.*

| | | |
|---|---|---|
| Acute dropsy from exposure. Dropsy after scarlet fever. | Urine deep colored, of high specific gravity, containing much albumen, often blood; also fibrin-casts, covered with epithelium. | Kidney enlarged and vascular, shedding its epithelium. |

B.

*Cases in which dropsy is variable in amount, chronic, and may be absent.*

| | | | |
|---|---|---|---|
| Fatty disease. (Bright's kidney.) | Persistent and obstinate dropsy, coming on gradually; face pale and puffed. | Urine contains much albumen, fatty casts, fat cells, free oil. Sp. gr. rather high, usually between 1015, 1030, rarely 1010. Quantity moderate or diminished. | Kidney in a state of chronic enlargement, mottled, fatty. |

| | | | |
|---|---|---|---|
| Waxy disease. | Dropsy trifling or entirely absent, great emaciation, great sallowness of face. Liver and spleen enlarged. | Urine contains much albumen, but only a few casts, which are pale and transparent. Sp. gr. varying, usually above 1010. | Kidney enlarged, smooth and waxy-looking. |
| Chronic nephritis, or chronic wasting of kidney. (Gouty kidney.) | Dropsy moderate, less than in fatty kidney. Face sallow, yet not so much so as in the waxy disease. Retention of urea, tendency to coma and to convulsions, impoverished blood. Liver may be cirrhosed. Gouty diathesis. | Urine more copious than in health, yet extremely small amount of albumen. Large granular casts, altered epithelium, a little oil. Sp. gr. very low, rarely above 1010, much oftener below. | Kidney small, dense, and contracted. |

Most of the same indications arise in the treatment of chronic renal dropsies, as in the acute, hence the same remedies are more or less used. The depraved state of the blood, and the tendency to retention of poisonous ingredients in the circulating fluid, must be constantly and especially borne in mind, and agents employed for their relief. Cerebral symptoms are best treated by counter irritations to the back of the neck or to the scalp, by free purging, and by using mild diuretics to promote the action of the kidney. Amongst the diuretics, Dr. Todd mentions favorably the benzoate of ammonia. We do not find in any of his cases of uræmia, that he has made use of the mineral acids, so strongly recommended by Frerichs.

Dropsies are far from being always associated with renal disease. Derangements of the circulation of any part; depression of its nervous power and nutrition, as in paralytic limbs; pressure on large venous trunks; a watery state of the blood, with an enfeebled and inactive capillary circulation, or the reverse one, gorged by inflammatory irritation, may all be productive of effusion of fluid.

To the dropsies from deranged circulation belongs one of the most troublesome of all dropsies, that connected with *disease of the heart.* Hypertrophy or dilatation, in all their different forms, with or without valvular disease, may be accompanied by dropsy. Of these conditions, dilatation of the right side of the heart, with disease of the tricuspid orifice permitting regurgitation, is the one most productive of effusions. There is then very difficult, at times spasmodic breathing, palpitation of the heart, congestion of the whole venous system, and regurgitation into the jugular vein, occasioning pulsation, or, at least, keeping the vessel full, even below a point pressed on with the finger. The dropsy commences in the extremities; anæmia is not unusually superadded,—a condition which still further favors the tendency to watery exudation. Under these circumstances, it is easy to see how dropsy is produced; not so easy is it where the valves on the left side are affected. Dr. Todd solves the difficulty by assuming that in *all* cases dilatation of the right side of the heart must necessarily occur before dropsy can be produced.

When dropsy occurs in disease of the heart, it is always a grave symptom; and yields but rarely to diuretics, or even to punctures or incisions employed for its relief. Especially is it grave, if accompanied by signs of disease of the kidney. The combination of lesions of these two organs is, unfortunately, a frequent clinical phenomenon. In an instructive paper published by Bamberger (Virchow's Archiv. Jan. 1857), the heart was found to be affected twenty-five times in forty cases of Bright's disease. The different parts, the endocardium, the pericardium, the muscles themselves, were all diseased in various degrees; but by far the most constant and marked pathological appearance was hypertrophy and dilatation, which occurred in nineteen cases, and in thirteen out of the nineteen on both sides of the heart.

Another great viscus, *the liver*, may be diseased when kidney and heart are affected, and thus add to the dropsy. The form of dropsy associated with alterations in the liver is ascites, or dropsy of the peritoneum. All chronic diseases of the liver can lead to ascites; but the one which especially does so is the granular liver, or cirrhosis,—the liver of persons of intemperate habits. Most of the continental writers, and, if we mistake not, Dr. Budd does the same, describe a state of enlargement of the liver as preceding the small, hard, contracted liver of cirrhosis. Dr. Todd suggests that these two states are different diseases, similar to the enlarged and contracted kidney, both producing, however, the same effect,—dropsy; because in both there is thickening of the capsule of Glisson, which, by retarding the portal circulation, furnishes a condition most favorable to effusion. One of the cases he cites, in which dropsy with enlargement and induration of the liver existed, is interesting on account of the good therapeutic results obtained. The use of frictions with strong mercurial ointment and equal parts of compound iodine ointment, of pressure on the abdomen by means of a bandage, and the internal administration of digitalis, squills, and mercury, removed the effusion completely in the space of seven weeks. In another case of ascites, not however connected with disease of the liver, but dependent probably on subacute peritonitis, a similar plan of treatment by mercurializing the patient, employing mercurial frictions, and diuretics, resulted in discharging, on the 2d of June, without any trace of swelling, a patient, admitted May 12th, measuring thirty-three inches round at the level of the umbilicus. Amongst the most useful diuretics, Dr. Todd mentions the bitartrate of potassa, lemon-juice, and the benzoate of ammonia, in doses from five to fifteen grains dissolved in water.

Many, however, are the cases of ascites, in which no remedies seem to avail. The dropsy increases, and the patient dies worn out. There is yet another termination, the rarity of which is much to be regretted. Nature may step in, and effect spontaneously what art has been vainly striving to effect. Medical literature furnishes a number of such unexpected and sudden terminations of ascites. We need hardly cite the well-known case of Dr. Graves, in which the patient passed off a dropsy of thirteen years standing, by means of her kidneys, in about a fortnight, causing the integuments of the abdomen, relieved from their previous enormous distension, to hang pendulous. M. Mondière (Journal l'Experience, tom. vii) has placed one as singular on record. Thirty pints were excreted from the kidney in seventy-two hours, and the ascites vanished. Dropsies have as suddenly disappeared by vomiting, by excessive discharges from the bowels, or by profuse sweating.

If medical means fail, and nature does not come to the relief, the operation of tapping alone affords a way of getting rid of the fluid; but not without danger, and not without a very strong probability of the effusion returning. Our author believes that tapping may be advised with better prospects of success, where the superficial veins are dilated and where the dropsy is connected with enlargement of the liver, than when it is with contraction. He directs the operation to be performed whilst the patient is under the effect of opium, and in a *horizontal* position, thus avoiding the tendency to syncope, and dispensing with a compressing bandage. Several cases are mentioned where tapping has resulted in an ultimate cure, after the operation had been once or several times resorted to. That there are scarcely any limits to its performance, but that of death, we know from the inscription engraved on the monument of good old Dame Page, handed down to us by Dr. Mead:

"Here lies Dame Mary Page,
Relict of Sir Gregory Page, Bart.,
She departed this life, March 4th, 1728,
In the 56th year of her age.
In 67 months she was tapped 66 times.
Had taken away 240 gallons of water," &c.

The four last of these clinical lectures are devoted to the appearance of pus in the urine, to gout in the bladder, and to a consideration of gout in general. Urine containing *pus* is muddy, when first passed. On standing, a whitish deposit falls to the bottom of the vessel, which shaking up with liquor potassa gelatinizes; treated with acids it becomes slightly more dense. In this fact we have a valuable differential test for pus. Phosphates also furnish a whitish deposit, but they are dissolved in acids; mucus is nearly soluble in acetic acid. The sources of pus in the urine are many. It may come from the kidney, from the ureter, from the bladder, from the urethra, or, in females, from the vagina; or, it may be derived from abscesses opening into the urinary passages. The character of the pus and of the urine does not, unfortunately, aid us much in determining its source. If from an irritated or diseased state of mucous membrane of the bladder, it is apt to contain triple phosphates. Pus is most frequently derived from this viscus; the irritation of retained urine, of a stone, simple cystitis, or the morbid state of the mucous membrane in gouty diathesis, will all produce it. Dr. Todd cites several interesting cases in which the symptoms pointed to gouty inflammation of the bladder. In one patient (Case XLIV), who was liable to attacks of shifting gout, the disease recurred annually, accompanied with pain in the left lumbar region, and pain in the bladder more severe than that in the back and antecedent to it. The urine was of a low specific gravity, alkaline, and contained blood and pus. In many cases, Dr. Todd remarks, this state of urine exists, with an impairment of the power of retaining urine, and is decidedly an inflammatory condition; in others, gout attacking the bladder may only give rise to great pain and distress after indiscretions in diet, speedily relieved by appropriate treatment; or, again, there may be incontinence of urine, the urine being pale and acid, or else an inability to pass water with distension of the bladder may be brought on, causing great suffering. Such are the four different ways in which gout may affect the bladder. The diagnosis is determined by the suddenness of the attack, the gouty diathesis, and the absence of other causes, such as renal or vesical calculi, to account for the symptoms.

In the asthenic and shifting gout there is a much greater liability to these attacks of internal organs. Even sthenic gout, Dr. Todd believes, is apt to attack the bladder, stomach, or heart, if treated by depressing means. The following well-told case is cited in support of this view:

"A friend of mine, of very robust make, but overworked in laborious professional employment, had a severe attack of gout in one foot. He had previously suffered from it on many occasions. Being much in request in his profession, he was impatient to get well, and, contrary to his better judgment, allowed a number of leeches to be applied to the gouty foot. The leech-bites bled freely, and next day he found that the active gout had quitted that foot, leaving it very weak, and had settled in the other. This latter swelled up quickly, and so intense was the inflammation that a neighboring surgeon, thinking that matter had formed, made an incision, which bled freely. On the following day, both feet were free from gout, but the bladder had become irritable, micturition was painful and frequent, and the urine contained bloody mucus. The vesical irritation yielded in a few days, and now the action of the heart became frightfully irregular and intermittent; and, notwithstanding that the gout was on more than one occasion successfully invited to the extremities, it was many months before the heart's action was restored in strength and rhythm."

The treatment in cases of gout of the bladder consists in free counter irritation by means of mustard, by ammonia, and in relieving pain by opiate injections; the action of the skin and bowels must be kept up, and, provided the urine be acid, alkalies will be found of service. In cases of internal gout affecting other organs, as the stomach or bronchial tubes, Dr. Todd recommends the same treatment by free counter-irritation; in gouty states of the stomach, stimulants, as brandy, ammonia, must be freely and fearlessly administered. Where the joints are much affected, a *small* blister is placed over them, and internally alkalies are prescribed. The use of leeches is deprecated, as a matter of experience; nor does the internal administration of that much-vaunted, much-employed, and, on the other hand, much-abused remedy, *colchicum,* find an advocate in the distinguished physician of King's College Hospital.

